# Factors Affecting Best-Tolerated Dose of Pirfenidone in Patients with Fibrosing Interstitial Lung Disease

**DOI:** 10.3390/jcm12206513

**Published:** 2023-10-13

**Authors:** Neha P. Mandovra, Preyas J. Vaidya, Ria S. Shah, Aishwarya S. Nighojkar, Vinod B. Chavhan, Ayush Lohiya, Joerg D. Leuppi, Anne Leuppi-Taegtmeyer, Prashant N. Chhajed

**Affiliations:** 1 Institute of Pulmonology Medical Research and Development, Mumbai 400054, India; drnehamandovra@gmail.com (N.P.M.); preyas2@gmail.com (P.J.V.); drria.s21@gmail.com (R.S.S.); aishwaryanighojkar@outlook.com (A.S.N.); drvinodbchavhan@gmail.com (V.B.C.); 2 Department of Respiratory Medicine, Fortis Hiranandani Hospital, Navi Mumbai 400614, India; 3 Kalyan Singh Super Speciality Cancer Institute, Lucknow 226002, India; ayush.025@gmail.com; 4 University Centre of Internal Medicine, Kantonsspital Baselland, 4410 Liestal, Switzerland; joerg.leuppi@ksbl.ch (J.D.L.); anne.leuppi-taegtmeyer@usb.ch (A.L.-T.); 5 Medical Faculty, University of Basel, 4031 Basel, Switzerland; 6 Department of Patient Safety, Medical Directorate, University Hospital Basel, 4031 Basel, Switzerland

**Keywords:** anti-fibrotics, ILD, nintedanib, pirfenidone

## Abstract

The aim of the study was to examine the best-tolerated dose of pirfenidone, the adverse effects profile, and potential factors other than drug dose influencing the tolerability of pirfenidone in patients with fibrosing interstitial lung diseases (ILDs). We performed an observational retrospective study of 113 patients with IPF and other fibrosing ILDs treated with pirfenidone. Baseline liver function tests (LFTs) and dose escalation of pirfenidone were recorded for all patients. The best-tolerated dose was continued if the patient did not tolerate full dose (2400 mg) despite repeated dose escalation attempts. Potential risk factors such as age, height, weight, body mass index (BMI), body surface area (BSA), gender, smoking, and presence of comorbidities were analyzed between 3 groups of best-tolerated pirfenidone doses: 2400 mg/day vs. <2400 mg/day, 2400 mg/day vs. 1800 mg/day, and 2400 mg/day vs. 1200 mg/day. A total of 24 patients tolerated 2400 mg/day, and 89 patients tolerated <2400 mg/day (43 tolerated 1800 mg/day, 45 tolerated 1200 mg/day and 1 tolerated 600 mg/day). Patients who tolerated 2400 mg/day were taller and had a larger BSA as compared to those tolerating <2400 mg/day. Overall, males tolerated the drug better. Presence of comorbidities or smoking did not affect the tolerance of pirfenidone, except for the presence of cerebrovascular diseases. Various adverse effects did not have any significantly different frequencies between the compared groups. Moreover, 71.7% of patients experienced at least one side effect. 1200 mg/day was the best-tolerated dose in the majority of the patients. Male patients with a larger BSA and greater height showed better tolerability of pirfenidone overall.

## 1. Introduction

Idiopathic pulmonary fibrosis (IPF) is a chronic, progressive, and fatal fibrosing lung disorder of unknown etiology leading to scarring of the pulmonary parenchyma and a decline in lung capacity, leading to respiratory failure and death within 2 to 5 years of diagnosis in patients not receiving anti-fibrotic therapy [1,2,3]. Pirfenidone and nintedanib are the two anti-fibrotic agents approved for the treatment of IPF [4]. Both pirfenidone and nintedanib have shown a reduction in decline in forced vital capacity (FVC) with favourable adverse effect profile and tolerability in various randomised clinical trials [5,6,7,8,9]. Pirfenidone has also been shown to reduce the decline in exercise capacity as defined by six-minute walk distance (6MWD) and progression-free survival.

Pirfenidone was the first anti-fibrotic drug approved for the treatment of IPF. It has shown anti-fibrotic and anti-inflammatory effects in both in vitro and in vivo studies [10,11]. It acts by decreasing the synthesis and accumulation of collagen in lung tissue, downregulating the pulmonary growth factor transforming growth factor-B1, and preventing the expression of intracellular adhesion molecule-1, which plays an important role in the development of fibrosis [12]. Pirfenidone is licensed to be given at a maximum dose of 801 mg three times daily (2403 mg/day, the recommended dose) [13]. According to the product information, patients who experience significant adverse reactions should receive reduced doses of pirfenidone. The efficacy of reduced pirfenidone doses compared to placebo has been demonstrated in a post-hoc analysis of the three phase III licensing trials of pirfenidone in IPF [14]. These findings support continuing pirfenidone at a reduced dose in patients with IPD.

In a study integrating data from five clinical studies, it was observed that pirfenidone was safe and generally well tolerated; however, nearly all patients experienced at least one side effect [15]. The most common side effects observed were gastrointestinal side effects and skin-related events [15]. The Capacity trials have also shown the benefits of administering the full recommended dose of pirfenidone [8]. However, due to the occurrence of side effects, it has been reported that the full dose of pirfenidone (2403 mg) may not be tolerated by all patients and may require dose modification or dose reduction to maintain adherence [14,16]. We also observed that in our clinical setting, not all patients tolerated the full recommended dose of pirfenidone. In times when only one anti-fibrotic drug was available for the treatment of IPF, if the patient did not tolerate the full dose of pirfenidone, then whatever lower dose was tolerated was administered for whatever benefit.

Some years ago, nintedanib became the second drug to be approved for the treatment of IPF [7]. So far, no trials have conclusively shown the benefit of one anti-fibrotic over the other in the treatment of IPF. Therefore, when a patient does not tolerate one anti-fibrotic drug, the second automatically needs to be considered. The characteristics of patients developing gastrointestinal side effects with nintedanib and risk factors leading to the development of side effects have been reported recently [17]. In our setting at the time of this study, pirfenidone was available as a generic drug and hence at a lower cost than nintedanib, which was then available as a research molecule and at a very high cost. Therefore, in our setting, pirfenidone was typically the preferred anti-fibrotic over nintedanib as most of the patients did not have re-imbursement and needed to pay out of pocket.

With the approval of nintedanib as an anti-fibrotic treatment for IPF, in our setting too, it is considered an alternative treatment option when the patient does not tolerate the full dose of pirfenidone. To the best of our knowledge, potential factors influencing the dose of pirfenidone in patients with interstitial lung disease have not been studied. We undertook the current study to examine the best-tolerated dose of pirfenidone in our clinical setting, the profile of adverse effects of pirfenidone in our population, and the potential risk factors other than adverse effects influencing the best-tolerated dose of pirfenidone.

## 2. Methods

This was an observational, retrospective study including patients with IPF and other fibrosing interstitial lung diseases treated with pirfenidone. Pirfenidone was also used off-label in other fibrosing ILDs in whom progression occurred despite maximal optimal therapy and in patients with H1N1 lung fibrosis [18,19,20]. Records of patients who were treated over a period of four years in a respiratory outpatient clinic specializing in ILD were reviewed. Diagnosis of IPF was made by using official clinical recommendations for the diagnosis of IPF by ATS/ERS/JRS/ALAT and the Indian guidelines for the diagnosis of IPF [1,21].

As a typical practice in our setting, options for both pirfenidone and nintedanib are discussed in detail, including the adverse effects related to both drugs, the pill burden associated with pirfenidone, and the cost differential of both drugs, with patients and their families. All patients included in the study made an informed choice about the initiation of pirfenidone.

Pirfenidone was started at a dose of 600 mg three times a day, and the dose was increased by 600 mg per day every one to two weeks with monitoring of liver function tests and other side effects until a dose of 2400 mg per day was achieved. Dose escalation of pirfenidone was discontinued in the case of side effects. Patients were advised to take pirfenidone with meals in order to reduce the gastrointestinal side effects and improve tolerability [22]. If the patient did not tolerate the full dose despite dose escalation attempts every two to four weeks, a lower but best-tolerated dose was continued. Smokers were instructed to stop smoking.

A stepwise management strategy was followed for preventing and alleviating gastrointestinal and skin-related side effects [23,24]. Liver function test was monitored every 2 weeks. Elevations of aspartate transaminase (AST) and alanine transaminase (ALT) levels to >3× the upper limit of normal (ULN) were managed by dose modifications or discontinuation. If AST and ALT elevations (>3× to ≤5× ULN) occurred without symptoms or hyperbilirubinaemia, the dose was reduced or interrupted until values return to normal. If the AST and ALT elevations (>3× to ≤5× ULN) were accompanied by hyperbilirubinaemia, pirfenidone was permanently discontinued. If patients exhibited >5× ULN, pirfenidone was permanently discontinued. These tests were conducted before the initiation of pirfenidone treatment, at monthly intervals for the first 6 months, and then monitored every 3 months thereafter.

Patients were encouraged to reduce sun exposure through the use of sunscreen and appropriate clothing during pirfenidone use. In cases of severe photosensitivity and rash, patients were given topical silver sulfadiazine or steroids. An echocardiogram was also ordered. Patients were also encouraged to stay well hydrated. Patients who experienced a loss of appetite had their eating habits discussed, and they were counselled to take small, portioned meals frequently. In patients who had nausea, acid peptic symptoms, or gastroesophageal reflux, proton pump inhibitors and H2 (histamine) blockers were advised. Patients who experienced weight loss were encouraged to eat fatty meals along with dietary supplements, increasing the frequency and size of meals. For patients who did not improve from the above measures, dose reduction and drug holidays were given.

In the current study, the records of 177 patients who were initiated on pirfenidone were reviewed. Of these, 64 patients came for a second opinion and were referred back to their primary physician; hence, they were excluded from the study due to a lack of follow-up data.

Details of the initial and follow-up visits after 2 weeks, 3 months, and 6 months were recorded. Any dose reduction made due to side effects, type of side effects, and the best-tolerated dose of pirfenidone were noted. Various potential risk factors such as age, gender, height, weight, body mass index (BMI), body surface area (BSA), smoking, and the presence of comorbidities were analysed. Baseline liver function test was noted. Other details included a history regarding any adverse effects due to pirfenidone, symptomatic treatment given for the same, a baseline pulmonary function test, diffusion capacity, and a six-minute walk test. Echocardiography, which was performed to assess the presence of pulmonary hypertension, was recorded.

### Statistical Analysis

Descriptive analysis was performed, and categorical variables were described as frequencies with percentages. Continuous variables were expressed as means with standard deviation (SD). Bivariate analysis was performed to delineate the factors associated with “dose of tolerance” (maximum tolerated dose). Three comparisons were done to identify the factors associated with the best-tolerated dose of pirfenidone: (1) 2400 mg/day vs. <2400 mg/day, (2) 2400 mg/day vs. 1200 mg/day, and (3) 2400 mg/day vs. 1800 mg/day. For the comparison of categorical variables, the chi-square test was applied, and odds ratios were calculated. Continuous variables were compared using Student’s *t*-test. Binary logistic regression was performed to identify the independent predictors of the best-tolerated dose of pirfenidone (2400 mg/day vs. <2400 mg/day). A *p*-value of <0.05 was considered statistically significant. The analysis was performed in Statistical Package for Social Sciences (IBM Corp., released 2011). IBM SPSS Statistics for Windows, Version 20.0. IBM Corp., Armonk, NY, USA). The study was approved by the ethics committee of Fortis Hiranandani Hospital, Vashi, India.

## 3. Results

A total of one hundred thirteen patients (42% females, mean age (SD) 65.5 (11) years) were included in the study. Baseline characteristics and demographics of the study population have been elaborated on in Table 1. Pirfenidone was administered in patients with IPF (n = 90, 79.6%) and also in certain non-IPF conditions having Fibrosing ILD (n = 23, 20.4%), including chronic hypersensitivity pneumonitis (n = 8, 7%), nonspecific interstitial pneumonias (NSIP) (n = 2, 1.8%), combined pulmonary fibrosis with emphysema (n = 6, 5.3%), post-H1N1 fibrosis (n = 2, 1.8%), rheumatoid arthritis-related ILD (n = 3, 2.7%), familial fibrosis (n = 1, 0.9%), and sarcoidosis (n = 1, 0.9%). The mean duration of follow-up was 505 days. The mean duration of administration of pirfenidone was 622 days (in some patients pirfenidone was started prior to the first visit to our centre). Table 2 outlines the various comorbid conditions present in the study population. Hypertension (36.3%), diabetes (32.7%), and secondary pulmonary hypertension (31.9%) were the most common comorbid conditions present in the study population. Side effects were observed in 81 out of 113 patients (71.7%). Common adverse effects manifested during the treatment in the study population were acid peptic symptoms (n = 44, 38.9%) and weight loss (n = 22, 19.5%), followed by loss of appetite (n = 18, 16%), transaminitis (n = 11, 9.7%), itching (n = 10, 8.8%), dizziness (n = 2, 1.8%), rash (n = 2, 1.8%), skin dryness (n = 1, 0.9%), burning in hands and feet (n = 1, 0.9%), skin hyperpigmentation (n = 1, 0.9%), tremors (n = 1, 0.9%), and stomatitis (n = 1, 0.9%) (Table 3). The dose was reduced in 89 out of 113 patients (78.8%), while 81 patients experienced side effects. In the remaining 8 patients, the dose was reduced due to the associated pill burden of pirfenidone (pirfenidone was available in the strength of 200 mg at the time of the study).

Comparison between 2400 mg/day (recommended dose) and <2400 mg/day of pirfenidone:

A total of 24 patients tolerated 2400 mg/day and 89 patients tolerated <2400 mg/day, out of which 43 tolerated 1800 mg/day, 45 tolerated 1200 mg/day, and one patient tolerated 600 mg/day of pirfenidone. There was no significant difference in age, weight, BMI, baseline FVC% predicted, or baseline FEV_1_% predicted between those who tolerated 2400 mg and those who tolerated <2400 mg (Table 3). However, there was a statistically significant difference in height and BSA between the two groups, with height and BSA being higher in those tolerating the full 2400 mg dose (Table 4). Likewise, the mean BSA-adjusted daily dose was significantly higher in those tolerating the 2400 mg dose (*p* < 0.001). Males tolerated the drug better as compared to females (*p*-value < 0.004) (Table 5). The presence of various comorbidities did not affect the tolerance of pirfenidone, except for the patients who had an incidence of cerebrovascular diseases, who had a better tolerance of pirfenidone (Table 5). The occurrence of various adverse effects did not show any significant difference between the two groups (Table 5).

Multivariate analysis revealed that weight (*p* = 0.016) and presence of cerebrovascular disease (*p* = 0.019) were significantly different between those who were taking 2400 and <2400 mg pirfenidone.

Comparison between 2400 mg/day (recommended dose) and 1200 mg/day of pirfenidone: Similar results were reported with significantly higher height and BSA in the 2400 mg/day group (Table 6), with better tolerability in males (Table 7). No significant difference was observed in adverse effects between the two groups except for itching, which was observed more in patients tolerating 1200 mg/day (Table 7).

Comparison between 2400 mg/day (recommended dose) and 1800 mg/day of pirfenidone:

Patients who tolerated 2400 mg/day had significantly higher weights as compared to patients who tolerated 1800 mg/day. BSA was also significantly higher in the 2400 mg/day group (Table 8). Males tolerated the drug better in the 2400 mg/day group as compared with the 1800 mg/day group, and the presence of comorbidity did not influence the tolerance of pirfenidone between the groups except for the presence of cerebrovascular diseases (Table 9).

Eleven patients receiving 2400 mg/day developed deranged liver enzymes, which eventually improved after reducing the dose to 1800 mg/day in 7 patients and to 1200 mg/day in 4 patients. Fourteen of 113 patients discontinued pirfenidone (12.4%), 8 due to intolerability (7.07%), and 6 due to disease progression (5.3%). Nintedanib was initiated in 6 patients with disease progression.

## 4. Discussion

Pirfenidone is the first of the two anti-fibrotic drugs approved for the treatment of IPF. Controlled clinical trials have shown significant benefits in halting disease progression and possibly reducing mortality [8]. To the best of our knowledge, the current study is the first to examine the best-tolerated dose of pirfenidone and to assess the potential risk factors influencing the best-tolerated dose other than adverse effects.

The main findings of our study were that not all patients tolerated the full recommended dose of pirfenidone due to the occurrence of side effects. 71.7% of patients experienced at least one side effect. The frequency of occurrence of side effects in the current study was similar to the findings of the RECAP trial, which reported an incidence of 74.3% [25]. The most frequent side effects observed were acid peptic symptoms such as nausea and vomiting (38.9%) and weight loss (19.5%), which have been similarly reported in other studies [14]. However, whether weight loss was due to pirfenidone or disease progression could not be separated. The RECAP trial showed a discontinuation rate for pirfenidone of 33.8%, compared to 12.4% in the current study population [25]. This is likely attributed to the fact that patients in the current study were permitted to continue at a dose lower than 2400 mg/day.

The objective of the current study was to determine whether any other risk factor influenced the best-tolerated dose of pirfenidone in the current population. In the current study, we found that males tolerated pirfenidone better than females. Similar findings have been observed in two other studies [14,26].

A higher dose of pirfenidone was tolerated better by patients who were taller and had a higher BSA. This could be explained by pirfenidone’s narrow therapeutic index and high protein binding. Therefore, patients with a lower BSA are less likely to tolerate the full recommended dose of 2400 mg/day [27]. A Japanese study in patients with IPF has suggested that a BSA-related dose adjustment of pirfenidone could be adequate to prevent side effects and still achieve effective treatment [28]. Smoking and the presence of various comorbidities did not influence the best-tolerated dose of pirfenidone.

The limitations of the current study include its observational and retrospective nature. Nevertheless, it provides real-life data from a single clinical centre. Being a referral tertiary care centre, many patients who were diagnosed with IPF and were initiated on pirfenidone were referred back to their primary treating physician with a plan of gradual escalation of the pirfenidone dose with monitoring of liver function tests. Hence, details of such patients were not available thereafter, thereby excluding them from the analysis. With time, some of the patients with advanced lung disease could not reach our centre for follow-up for various reasons, including those on home oxygen therapy who could not organise transportable oxygen and the eventual progressive nature of the disease confining them indoors. Therefore, we did not examine outcome parameters such as mortality and focussed on the best-tolerated dose of pirfenidone in our patient population. Only the baseline body weight, BMI, and BSA were analysed. The change in body weight, BMI, and BSA over time could not be analysed. The impact of various dosages of pirfenidone on disease progression (as indicated by serial FVC measurements) and mortality was also beyond the scope of the current study. Further studies may be needed to evaluate the influence of various doses of pirfenidone on measures of disease outcome.

## 5. Conclusions

A significant number of patients tolerated less than the recommended dose of pirfenidone. Based on the available reports, this has an important potential implication on disease progression [5,8,25] and the need to consider the second available antifibrotic, nintedanib, versus continuing a suboptimal dose of pirfenidone. The recommended dose of pirfenidone is not tolerated by all patients with fibrosing ILD.

## Figures and Tables

**Table 1:** Demographics and baseline characteristics of the study population.

Characteristics	Values ^a^ (n = 113)
Females, n (%)	48 (42.0)
Age in years (SD)	65.5 (11.0)
Weight in kg (SD)	62.7 (11.8)
Height ^b^ in cm (SD) (n = 87)	159 (9.8)
BMI ^c^ in kg/m^2^ (SD)	24.9 (4.4)
BSA ^d^ in m^2^ (SD)	1.64 (0.2)
FVC ^e^ in litres (SD)	1.54 (0.5)
FVC ^e^, % predicted (SD)	60.3 (16.4)
FEV_1_ ^f^ in litres (SD)	1.30 (0.48)
FEV_1_ ^f^, % predicted (SD)	64.2 (17)
Total smokers, n (%)	19 (17.0%)
Current smoker, n (%)	3 (2.6%)
Reformed smoker, n (%)	16 (14.0%)
Home oxygen, n (%)	42 (37.0%)
Duration of follow-up in days (SD)	505 (607.3)
Duration of pirfenidone received in days (SD)	622.2 (657.0)
Received additional steroid, n (%)	40 (35.4%)

6MWD, 6 min walk distance; BMI, body mass index; BSA, body surface area; DLCO, diffusion capacity for carbon monoxide; FVC, forced vital capacity; FEV_1_ forced expiratory volume in 1 s. ^a^ All values are mean (SD) unless otherwise noted; ^b^ n = 87 patients with available data; ^c^ n = 87 patients with available data; ^d^ n = 87 patients with available data; ^e^ n = 67 patients with available data; ^f^ n = 67 patients with available data.

**Table 2:** Comorbidities in the study population (n = 113).

Comorbidities	Number of Patients (%)
Hypertension	41 (36.3%)
Diabetes mellitus	37 (32.7%)
Ischemic heart disease	23 (20.4%)
Cerebrovascular disease	6 (5.3%)
Hypothyroidism	11 (9.7%)
Obstructive airway disease	22 (19.5%)
Past history of tuberculosis	6 (5.3%)
Osteoporosis	33 (29.2%)
Secondary pulmonary hypertension	36 (31.9%)
Malignancy	4 (3.5%)
Lung cancer	1 (0.9%)
Endometrial cancer	1 (0.9%)
Penile cancer	1 (0.9%)
Prostate cancer	1 (0.9%)
Parkinson’s disease	1 (0.9%)
Hepatitis B	1 (0.9%)
Ankylosing spondylitis	1 (0.9%)
Myasthenia gravis	1 (0.9%)
Lumbar radiculopathy	1 (0.9%)
Nephrotic syndrome	1 (0.9%)
Monoclonal gammopathy	1 (0.9%)
Seizure disorder	1 (0.9%)

**Table 3:** Various adverse effects in the study population.

Adverse Effects	Number of Patients (%)
Acid peptic symptoms	44 (38.9)
Weight loss	22 (19.5)
Loss of appetite	18 (16.0)
Elevated liver enzymes	11 (9.7)
Itching	10 (8.8)
Dizziness	2 (1.8)
Rash	2 (1.8)
Skin dryness	1 (0.9)
Burning in hands and feet	1 (0.9)
Skin hyperpigmentation	1 (0.9)
Tremors	1 (0.9)
Stomatitis	1 (0.9)

**Table 4:** Comparisons of morphological and functional characteristics between patients receiving 2400 mg and <2400 mg pirfenidone.

Factor	Group	Number of Patients (n)	Mean	SD	*p*-Value
Age (years)	2400 mg	24	64.5	9.0	0.513
	<2400 mg	89	66.2	11.6	
Weight (kg)	2400 mg	24	66.8	11.3	0.06
	<2400 mg	89	61.6	11.9	
Height (cm)	2400 mg	20	162.9	6.2	0.046
	<2400 mg	67	157.9	10.4	
BSA (m^2^)	2400 mg	20	1.7	0.1	0.018
	<2400 mg	67	1.6	0.2	
BMI (kg/m^2^)	2400 mg	20	25.4	4.5	0.52
	<2400 mg	67	24.7	4.4	
FVC, % predicted	2400 mg	13	56.5	17.1	0.36
	<2400 mg	54	61.2	16.5	
FEV_1_, % predicted	2400 mg	13	62.9	18.5	0.77
	<2400 mg	54	64.5	16.8	

BSA: Body Surface Area is the total surface area of the human body.

**Table 5:** Comparison of comorbidities and adverse effect profile between patients receiving 2400 mg and <2400 mg pirfenidone.

Factor		2400 mg, n (%)	<2400 mg, n (%)	*p*-Value	Odds Ratio (95% CI)
Gender	Male	20 (30.8)	45 (69.2)	0.004	4.9 (1.5–15.5)
	Female	4 (8.3)	44 (91.7)		
Smoking	Yes	4 (21.1)	15 (78.9)	0.98	0.99 (0.29–3.3)
	No	20 (21.3)	74 (78.7)		
Hypertension	Yes	8 (19.5)	33 (80.5)	0.74	0.85 (0.33–2.2)
	No	16 (22.2)	56 (77.8)		
Diabetes	Yes	8 (21.6)	29 (78.4)	0.95	1.03 (0.39–2.7)
	No	16 (21.1)	60 (78.9)		
Hypothyroidism	Yes	1 (9.1)	10 (90.9)	0.30	0.34 (0.4–2.8)
	No	23 (22.5)	79 (77.5)		
Obstructive airway disease	Yes	3 (13.6)	19 (86.4)	0.33	0.53 (0.14–1.9)
	No	21 (23.1)	70 (76.9)		
Cerebrovascular disease	Yes	5 (83.3)	1 (16.7)	<0.001	23.2 (2.6–209.7)
	No	19 (17.8)	88 (82.2)		
Ischemic heart disease	Yes	7 (30.4)	16 (69.6)	0.23	1.9 (0.67–5.3)
	No	17 (18.9)	73 (81.1)		
Osteoporosis	Yes	4 (12.1)	29 (87.9)	0.13	0.41 (0.13–1.32)
	No	20 (25.0)	60 (75.0)		
Secondarypulmonary hypertension	Yes	7 (19.4)	29 (80.6)	0.75	0.85 (0.32–2.28)
	No	17 (22.1)	60 (77.9)		
Loss of appetite	Yes	3 (16.7)	15 (83.3)	0.61	0.71 (0.19–2.7)
	No	21 (22.1)	74 (77.9)		
Weight loss	Yes	4 (18.2)	18 (81.8)	0.69	0.79 (0.24–2.6)
	No	20 (22.0)	71 (78.0)		
Elevated liver enzymes	Yes	0 (0.0)	11 (100.0)	0.07	0.76 (0.69–0.85)
	No	24 (23.5)	78 (76.5)		
Acid peptic symptoms	Yes	6 (13.6)	38 (86.4)	0.12	0.46 (0.16–1.23)
	No	18 (26.1)	51 (73.9)		
Itching	Yes	0 (0.0)	10 (100.0)	0.09	0.77 (0.69–0.85)
	No	24 (23.3)	79 (76.7)		
Skin dryness	Yes	1 (100.0)	0 (0.0)	0.05	0.2 (0.14–0.3)
	No	23 (20.5)	89 (79.5)		
Dizziness	Yes	0 (0.0)	2 (100.0)	0.46	0.78 (0.70–0.86)
	No	24 (21.6)	87 (78.4)		
Burning in hands and feet	Yes	0 (0.0)	1 (100.0)	0.60	0.79 (0.70–0.87)
	No	24 (21.4)	88 (78.6)		
Rash	Yes	0 (0.0)	2 (100.0)	0.46	0.78 (0.70–0.86)
	No	24 (21.6)	89 (78.8)		
Stomatitis	Yes	0 (0.0)	11 (100.0)	0.60	0.79 (0.71–0.87)
	No	24 (21.4)	88 (78.6)		
Hyperpigmentation	Yes	0 (0.0)	1 (100.0)	0.60	0.79 (0.71–0.87)
	No	24 (21.4)	88 (78.6)		
Tremors	Yes	0 (0)	1 (100)	0.60	0.79 (0.71–0.87)
	No	24 (21.4)	88 (78.6)		

**Table 6:** Comparisons of morphological and functional characteristics between patients receiving 2400 mg and 1200 mg pirfenidone.

Factor	Group	Number of Patients (n)	Mean	SD	*p*-Value
Age (years)	2400 mg	24	64.5	9.0	0.749
	1200 mg	45	63.6	13.1	
Weight (kg)	2400 mg	24	66.8	11.3	0.158
	1200 mg	45	62.0	14.1	
Height (cm)	2400 mg	20	162.9	6.2	0.036
	1200 mg	37	157.2	10.9	
BSA (m^2^)	2400 mg	20	1.7	0.1	0.029
	1200 mg	37	1.6	0.2	
BMI (kg/m^2^)	2400 mg	20	25.4	4.5	0.626
	1200 mg	37	24.8	4.6	
FVC, % predicted	2400 mg	13	56.5	17.1	0.52
	1200 mg	30	60.2	16.9	
FEV_1_, % predicted	2400 mg	13	62.9	18.5	0.94
	1200 mg	29	62.5	16.7	

BSA: Body Surface Area is the total surface area of the human body.

**Table 7:** Comparison of comorbidities and adverse effect profile between patients receiving 2400 mg and 1200 mg pirfenidone.

Factor		2400 mg, n (%)	1200 mg, n (%)	*p*-Value	Odds Ratio (95% CI)
Gender	Male	20 (45.5)	24 (54.5)	0.014	4.40 (1.28–14.86)
	Female	4 (16.0)	21 (84.0)		
Smoking	Yes	4 (28.6)	10 (71.4)	0.585	0.70 (0.19–2.50)
	No	20 (36.4)	35 (63.6)		
Hypertension	Yes	8 (34.8)	15 (65.2)	1	1.00 (0.35–2.86)
	No	16 (34.8)	30 (65.2)		
Diabetes	Yes	8 (42.1)	11 (57.9)	0.431	1.55 (0.52–4.58)
	No	16 (32.0)	34 (68.0)		
Hypothyroidism	Yes	1 (25.0)	3 (75.0)	0.672	0.61 (0.06–6.20)
	No	23 (35.4)	42 (64.6)		
Obstructive airway disease	Yes	3 (18.8)	13 (81.3)	0.124	0.35 (0.09–1.39)
	No	21 (39.6)	32 (60.4)		
Cerebrovascular disease	Yes	5 (83.3)	1 (16.7)	0.009	11.58 (1.27–105.90)
	No	19 (30.2)	44 (69.8)		
Ischemic heart disease	Yes	7 (58.3)	5 (41.7)	0.059	3.29 (0.92–11.90)
	No	17 (29.8)	40 (70.2)		
Osteoporosis	Yes	4 (23.5)	13 (76.5)	0.262	0.49 (0.14–1.70)
	No	20 (38.5)	32 (61.5)		
Secondary pulmonary hypertension	Yes	7 (31.8)	15 (68.2)	0.724	0.82 (0.28–2.40)
	No	17 (36.2)	30 (63.8)		
Loss of appetite	Yes	3 (30.0)	7 (70.0)	0.731	0.78 (0.18–3.32)
	No	21 (35.6)	38 (64.4)		
Weight loss	Yes	4 (28.6)	10 (71.4)	0.585	0.70 (0.19–2.52)
	No	20 (36.4)	35 (63.6)		
Elevated liver enzymes	Yes	0 (0.0)	4 (100.0)	0.132	0.63 (0.52–0.08)
	No	24 (36.9)	41 (63.1)		
Acid peptic symptoms	Yes	6 (23.1)	20 (76.9)	0.112	0.42 (0.14–1.24)
	No	18 (41.9)	25 (58.1)		
Itching	Yes	0 (0.0)	7 (100.0)	0.042	0.61 (0.50–0.75)
	No	24 (38.7)	38 (61.3)		
Skin dryness	Yes	1 (100.0)	0 (0.0)	0.168	0.34 (0.24–0.47)
	No	23 (33.8)	45 (66.2)		
Dizziness	Yes	0 (0.0)	0 (0.0)		
	No	24 (34.8)	45 (65.2)		
Burning in hands and feet	Yes	0 (0.0)	1 (100.0)	0.462	0.65 (0.54–0.77)
	No	24 (35.3)	44 (64.7)		
Rash	Yes	0 (0.0)	1 (100.0)	0.462	0.65 (0.54–0.77)
	No	24 (35.3)	44 (64.7)		
Stomatitis	Yes	0 (0.0)	0 (0.0)		
	No	24 (34.8)	45 (65.2)		
Hyperpigmentation	Yes	0 (0.0)	1 (100.0)	0.462	0.65 (0.54–0.77)
	No	24 (35.3)	44 (64.7)		
Tremors	Yes	0 (0.0)	1 (100.0)	0.462	0.65 (0.54–0.77)
	No	24 (35.3)	44 (64.7)		

**Table 8:** Comparisons of morphological and functional characteristics between patients receiving 2400 mg and 1800 mg pirfenidone.

Factor	Group	Number of Patients (n)	Mean	SD	*p*-Value
Age (years)	2400 mg	24	64.5	9.0	0.063
	1800 mg	43	68.9	9.2	
Weight (kg)	2400 mg	24	66.8	11.3	0.048
	1800 mg	43	61.8	8.7	
Height (cm)	2400 mg	20	162.9	6.2	0.134
	1800 mg	29	159.0	10.1	
BSA (m^2^)	2400 mg	20	1.7	0.1	0.033
	1800 mg	29	1.6	0.1	
BMI (kg/m^2^)	2400 mg	20	25.4	4.5	0.639
	1800 mg	29	24.9	3.9	
FVC, % predicted	2400 mg	13	56.5	17.1	0.23
	1800 mg	23	63.4	15.9	
FEV_1_, %predicted	2400 mg	13	62.9	18.5	0.46
	1800 mg	23	67.5	17.1	

FVC: Forced Vital Capacity is the volume of gas which is exhaled during a forced expiration starting from a position of full inspiration and ending at complete expiration.

**Table 9:** Comparison of comorbidities and adverse effect profile between patients receiving 2400 mg and 1800 mg pirfenidone.

Factor		2400 mg, n (%)	1800 mg, n (%)	*p*-Value	Odds Ratio (95% CI)
Gender	Male	20 (48.8)	21 (51.2)	0.005	0.19 (0.06–0.65)
	Female	4 (15.4)	22 (84.6)		
Smoking	Yes	4 (44.4)	5 (55.6)	0.562	0.66 (0.16–2.72)
	No	20 (34.5)	38 (65.5)		
Hypertension	Yes	8 (32.0)	17 (68.0)	0.615	1.3 (0.46–3.72)
	No	16 (38.1)	26 (61.9)		
Diabetes	Yes	8 (30.8)	18 (69.2)	0.492	1.44 (.51–4.1)
	No	16 (39.0)	25 (61.0)		
Hypothyroidism	Yes	1 (12.5)	7 (87.5)	0.143	4.47 (0.52–38.8)
	No	23 (39.0)	36 (61.0)		
Obstructive airway disease	Yes	3 (33.3)	6 (66.7)	0.867	0.88 (0.199–3.9)
	No	21 (36.2)	37 (63.8)		
Cerebrovascular disease	Yes	5 (100.0)	0 (0.0)	0.002	0.31 (0.21–0.45)
	No	19 (30.6)	43 (69.4)		
Ischemic heart disease	Yes	7 (38.9)	11 (61.1)	0.751	0.84 (0.27–2.54)
	No	17 (34.7)	32 (65.3)		
Osteoporosis	Yes	4 (20.0)	16 (80.0)	0.078	2.96 (0.86–10.2)
	No	20 (42.6)	27 (57.4)		
	No	17(36.2)	30 (63.8)		
Loss of appetite	Yes	3 (27.3)	8 (72.7)	0.518	0.63 (0.15–2.62)
	No	21 (37.5)	35 (62.5)		
Weight loss	Yes	4 (33.3)	8 (66.7)	0.843	0.86 (0.23–3.3)
	No	20 (36.4)	35 (63.6)		
Elevated liver enzymes	Yes	0 (0.0)	7 (100.0)	0.037	0.6 (0.49–0.74)
	No	24 (40.0)	36 (60.0)		
Acid peptic symptoms	Yes	6 (26.1)	17 (73.9)	0.23	0.51 (0.17–1.54)
	No	18 (40.9)	26 (59.1)		
Itching	Yes	0 (0.0)	3 (100.0)	0.186	0.63 (0.52–0.76)
	No	24 (37.5)	40 (62.5)		
Skin dryness	Yes	1 (100.0)	0 (0.0)	0.177	0.35 (0.25–0.49)
	No	23 (34.8)	43 (65.2)		
Dizziness	Yes	0 (0.0)	2 (100.0)	0.283	0.63 (0.52–0.76)
	No	24 (36.9)	41 (63.1)		
Burning in hands and feet	Yes	0 (0.0)	0 (0.0)		
	No	24 (35.8)	43 (64.2)		
Rash	Yes	0 (0.0)	1 (100.0)	0.452	0.64 (0.53–0.76)
	No	24 (36.4)	42 (63.6)		
Stomatitis	Yes	0 (0.0)	1 (100.0)	0.452	0.64 (0.53–0.76)
	No	24 (36.4)	43 (63.6)		
Hyperpigmentation	Yes	0 (0.0)	0 (0.0)		
	No	24 (35.8)	43 (64.2)		
Tremors	Yes	0 (0.0)	0 (0.0)		
	No	24 (35.8)	43 (64.2)		

## Data Availability

Data is contained within the current article.
